# Editorial on sartans and skeletal muscle regeneration: rethinking fibrosis as a modifiable target in traumatic injury

**DOI:** 10.1007/s00590-025-04446-7

**Published:** 2025-07-15

**Authors:** Filippo Migliorini, Raju Vaishya

**Affiliations:** 1 Department of Trauma and Reconstructive Surgery, University Hospital in Halle, Halle (Saale), Germany; 2 Department of Life Sciences, Health, and Health Professions, Link Campus University, Rome, Italy; 3 Department of Orthopaedic and Trauma Surgery, Academic Hospital of Bolzano (SABES-ASDAA), Bolzano, Italy; 4 Department of Orthopaedics and Joint Replacement Surgery, Indraprastha Apollo Hospitals, New Delhi, India

**Keywords:** Traumatic muscle injury, Fibrosis, Skeletal muscle regeneration, Antifibrotic therapy, Muscle repair, Translational medicine

## Abstract

Traumatic muscle injuries are common yet often lead to incomplete recovery, despite the high regenerative potential of skeletal muscle. A major obstacle is fibrosis, which replaces functional tissue with disorganised extracellular matrix, impairing contractility and healing. Central to this maladaptive response is the TGF-β1 pathway, a conserved fibrogenic signal also implicated in cardiac, hepatic, and renal fibrosis. This has prompted interest in repurposing antifibrotic agents, particularly angiotensin II receptor blockers (ARBs), or sartans. These drugs, widely used for hypertension, inhibit TGF-β1 activation via AT1R antagonism. Preclinical studies in murine models have shown that sartans reduce collagen deposition, promote muscle regeneration, and improve functional outcomes after injury. Some also activate additional regenerative pathways, such as PPAR-γ. Although no clinical trials have evaluated ARBs for muscle injuries, preliminary data from orthopaedic settings suggest potential benefits. Given their safety, availability, and biological plausibility, sartans represent a promising avenue for therapeutic modulation of fibrosis in muscle trauma. Future research should clarify optimal timing, dosing, and patient selection to translate these findings into clinical practice.

## Introductions

Traumatic muscle injuries remain a common yet underestimated problem in clinical practice. Whether resulting from high-energy trauma, surgical complications, or sports-related incidents, these injuries often leave patients with lasting deficits, reduced strength, limited range of motion, or chronic discomfort despite adequate mechanical repair. The paradox lies in the fact that skeletal muscle is one of the most regenerative tissues in the human body biologically. So why do so many patients fail to achieve full recovery? One key factor limiting successful muscle repair is the development of fibrosis. Rather than restoring contractile tissue, the post-injury environment often favours the deposition of a disorganised extracellular matrix. This fibrotic scar lacks function and mechanically interferes with muscle regeneration. The biological mechanisms underlying this maladaptive response are increasingly well understood; among them, the TGF-β1 pathway plays a central role. Perhaps more intriguing is that this pathway is not unique to muscle; it is conserved across several tissues affected by fibrosis, including the myocardium, liver, and kidney. This raises an important question: can drugs already employed to treat fibrosis in other organs be repurposed to benefit injured muscles? Among the most promising candidates are the angiotensin II receptor blockers (ARBs), also known as sartans. Initially developed to lower blood pressure, these agents have demonstrated the ability to interfere with fibrotic pathways beyond their cardiovascular effects. By antagonising the angiotensin II type 1 receptor (AT1R), sartans indirectly suppress TGF-β1 activation, thereby reducing the fibroblast-to-myofibroblast transition and subsequent collagen synthesis [1, 2]. This reduction in collagen synthesis is particularly significant as it allows for a more conducive environment for muscle regeneration, potentially enhancing the recovery process.

Preclinical data supporting the antifibrotic and pro-regenerative effects of sartans in skeletal muscle injury are steadily accumulating, particularly from murine models. One of the earliest and most widely cited studies in this field demonstrated that losartan, administered systemically after a muscle laceration injury in mice, resulted in a marked reduction of fibrotic tissue and improved functional recovery [1]. Histological analysis confirmed decreased collagen deposition and an increased cross-sectional area of regenerating myofibers. These improvements were linked to the suppression of TGF-β1 signalling, which is known to drive fibroblast-to-myofibroblast differentiation and matrix deposition. Subsequent studies have replicated and expanded upon these findings. In a model of cardiotoxin-induced injury, irbesartan and telmisartan both demonstrated similar reductions in fibrosis and enhanced expression of myogenic markers, such as MyoD and myogenin [2, 3]. In dystrophic muscle models, particularly the mdx mouse model of Duchenne muscular dystrophy, chronic administration of ARBs attenuated fibrosis and delayed the decline in muscle function [4]. These effects were attributed to both the suppression of canonical TGF-β signalling and, in the case of telmisartan, additional PPAR-γ activation, which likely contributed to anti-inflammatory and metabolic effects beneficial to the muscle environment. Beyond their effects on fibrosis, ARBs have also been shown to enhance vascular remodelling and reduce the infiltration of pro-inflammatory macrophages, further supporting a more regenerative milieu. Despite these promising results, the current evidence remains confined to animal models. No randomised clinical trials have been published to date assessing sartans as a therapeutic tool for acute traumatic muscle injuries in humans. Some exploratory work has investigated their use in orthopaedic surgery, for example, following ACL reconstruction or rotator cuff repair, where fibrosis around tendons or surgical planes is a known complication [6–8]. While preliminary results suggest improved healing profiles, the data remain sparse and insufficient to support clinical adoption.

Biology is complex. Skeletal muscle healing is not merely a binary between regeneration and fibrosis. In specific injury models, particularly those involving volumetric muscle loss, some matrix formation may be necessary to support force transmission or scaffold new tissue [9, 10]. In these contexts, indiscriminately reducing fibrosis could paradoxically destabilise the repair process. This emphasises the need for nuance: the timing of ARB administration, the injury's severity, and the host tissue's regenerative potential must all be considered when designing therapeutic strategies. Another aspect worth reflecting on is the translational gap. Despite the biological plausibility and the preclinical enthusiasm, we still lack solid clinical trials evaluating sartans for muscle injury in humans. A few exploratory studies have examined the use of ARBs in orthopaedic settings, such as tendon repair or ligament reconstruction; however, the evidence remains preliminary [6, 8]. Whether sartans can meaningfully improve outcomes in muscle-specific injuries or surgical scenarios with a high risk of fibrosis remains to be determined. At the same time, the safety and accessibility of ARBs make them particularly attractive. These are well-characterised molecules, widely prescribed, and relatively inexpensive. This could lower the barrier to future clinical implementation, especially in settings where fibrotic complications are predictable, such as post-operative care, sports injuries, or rehabilitation in older adults [11]. Perhaps the most significant conceptual value of exploring ARBs in muscle trauma lies in a shift of mindset. Historically, muscle injuries have been treated as mechanical problems: they tear, we repair; they rupture, we suture. However, the underlying biology is far richer and potentially more modifiable than previously appreciated. Pharmacological interventions targeting fibrosis, not merely inflammation, could redefine how we approach soft tissue healing [12]. This shift in mindset could inspire new approaches and strategies in the treatment of muscle trauma.

In conclusion, sartans emerge as a beacon of hope, offering a promising pharmacological tool to modulate the fibrotic response and potentially enhance regeneration following traumatic muscle injury. The evidence to date, though preclinical, paves the way for meaningful translational research. As we deepen our understanding of muscle biology, the idea that fibrosis is not an inevitable consequence but a modifiable outcome may open new and valuable avenues for patient care [13–15].

## Data Availability

No datasets were generated or analysed during the current study.
